# Sea urchin granuloma: A case of marine-related injury

**DOI:** 10.1016/j.jdcr.2025.06.063

**Published:** 2025-09-10

**Authors:** Sushmitha Dharani Sankar, Sheela Kuruvila, Karthiyaini Ashok, Sushmita Sreekumar

**Affiliations:** a Assistant Professor, Department of Dermatology, Aarupadai Veedu Medical College and Hospital, Puducherry, India; b Professor, Department of Dermatology, Aarupadai Veedu Medical College and Hospital, Puducherry, India; c Department of Dermatology, Aarupadai Veedu Medical College and Hospital, Puducherry, India

**Keywords:** foreign body reaction, marine injury, occupational dermatoses, sea urchin granuloma, Sea urchin injury, sea urchin sting

## Case description

**Fig 1:**
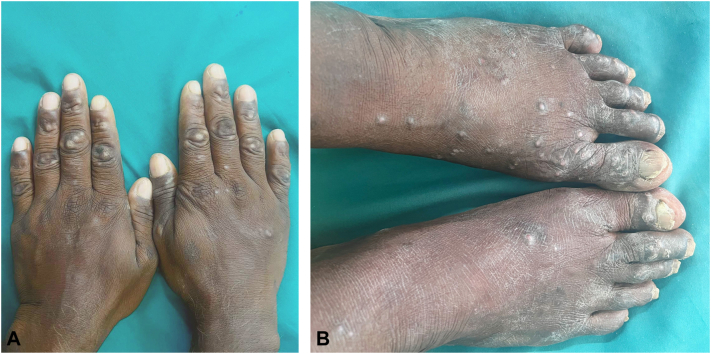
**(A, B)** Sea urchin granuloma presenting as multiple skin coloured to hyperpigmented nodules presenting over the dorsa of hands and feet.

**Fig 2:**
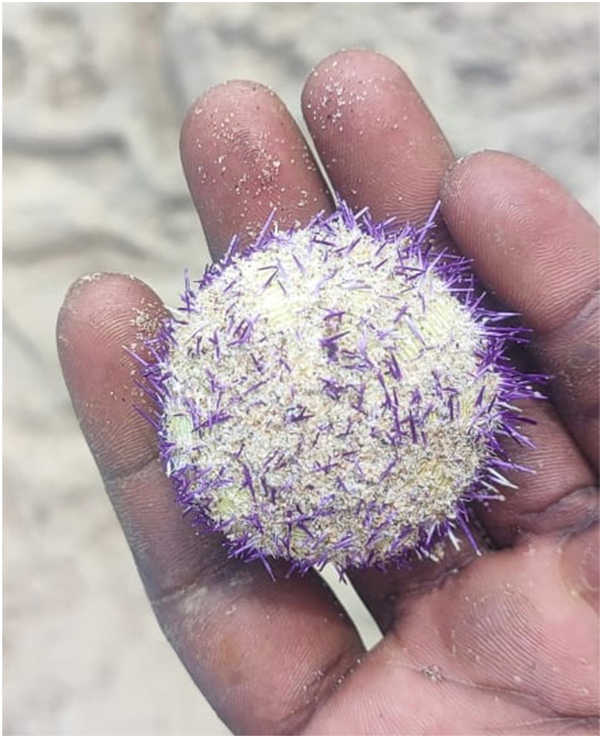
Sea urchin captured by the patient.

A 55-year-old fisherman presented with asymptomatic skin nodules on hands and feet for 2 months, after repeated exposure to marine organisms, including a recent incident 15 days prior. He gives a history of removing spines from lesions. Examination revealed multiple, well-defined skin-colored to hyperpigmented nodules over the dorsa of hands and feet (Fig 1, *A*). A 37-year-old fisherman presented with similar skin lesions over both hands for 1 month. The patient reported contact with sea urchins 4 months prior (Fig 2). Examination revealed multiple, well-defined hyperpigmented nodules over the dorsa of feet (Fig 1, *B*).

**Fig 3:**
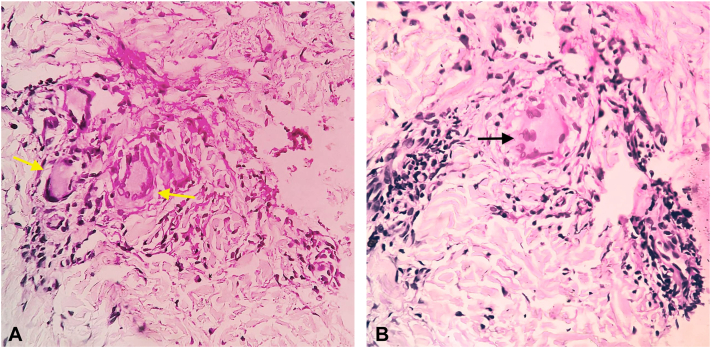
**(A, B)** H&E(40×) shows granulomas composed of Langhans giant cells (*yellow arrow*) and foreign body giant cells (*black arow*), admixed with lymphocytes and neutrophils.

Histopathology revealed well-defined epithelioid cell granulomas with Langhans giant cells (Fig 3, *A*). Notably, the second case also exhibited foreign body giant cells (Fig 3, *B*). Special stains for mycobacteria and fungi were negative.

**Question: What is the most probable diagnosis?**

1) Cutaneous tuberculosis
2) Sea urchin granuloma
3) Actinomycetoma
4) Atypical mycobacterial infection
5) Chromoblastomycosis

## Discussion

A diagnosis of sea urchin granuloma was made based on history, clinical findings, and histopathology. The patients were treated with topical steroids and oral doxycycline for broad-spectrum coverage, resulting in significant reduction of nodules within 2 months.

**Fig 4:**
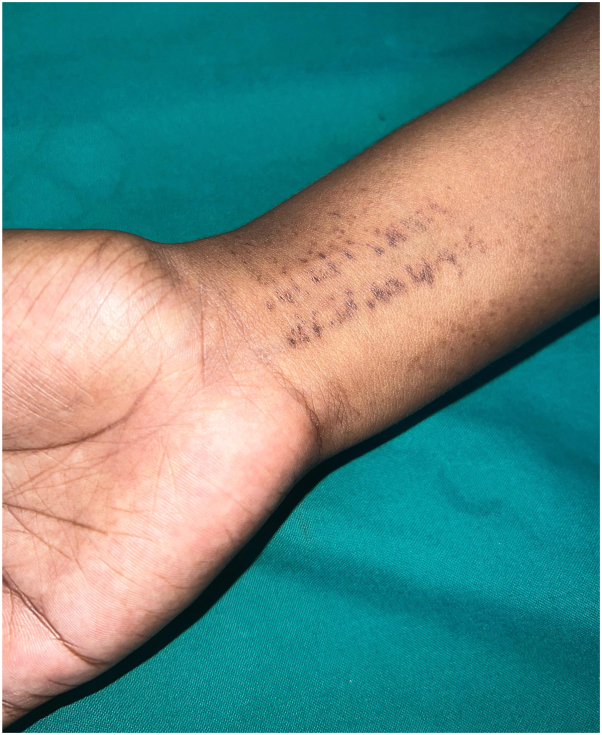
Post inflammatory pigmentation in a female patient secondary to contact with star fish.

Sea urchins, members of the phylum Echinodermata, are found in saltwater environments and can cause injuries with their spines. These spines contain bioactive substances that can trigger an immune response, leading to inflammation and pain.^1^ The clinical manifestations can vary, depending on the severity and nature of the injury. Minor injuries may cause localized, toxin-related effects, whereas extensive injuries involving multiple spines can cause severe systemic manifestations, including paresthesia, hypotension, paralysis, and respiratory distress and rarely, death.^2^ Delayed reactions usually occur at about 2 weeks post injury with manifestations of firm, pink to hyperpigmented nodules with edema and pain involving the extremities. Microscopically, sea urchin injuries may show foreign body reaction and granulomatous patterns, including sarcoidal, tuberculoid, or necrobiotic granulomas, or a mix of both.^3^^,^^4^ Secondary infections from sea urchin injuries can be caused by various organisms, including *Mycobacterium marinum*, *M. chelonae*, *Exophiala jeanselmei*, and marine-related bacteria like *Vibrio* species. Mycobacterial infections are a common cause of chronic joint complications in such cases. A study by De la Torre et al showed 21% of the sea urchin biopsy specimens showed evidence of mycobacterial infection by PCR.^3^^,^^5^ Hence, routine bacterial and mycobacterial cultures along with imaging like X-ray, ultrasound, or MRI may be of benefit in cases with clinical suspicion of infection and in delayed presentations with joint complications. Differentials include injuries from other echinoderms such as jellyfish, sea cucumbers, and starfish, as depicted here in another patient with post inflammatory patterned hyperpigmentation secondary to contact with starfish (Fig 4). Management involves swift removal of superficial spines manually or surgically, followed by warm water soaks and application of urea-based creams.^6^ Empiric antibiotics covering marine organisms are recommended; however, broad-spectrum antibiotics are only indicated if wound culture is positive or in immunosuppressed individuals. Treatment for mycobacterial and marine-related infections typically involves antibiotics such as cephalosporins, clindamycin, or cotrimoxazole with fluoroquinolones and tetracyclines for 2 weeks or until infection resolves.^6^ Doxycycline is preferred for its effectiveness against saltwater organisms and anti-inflammatory properties. Systemic steroids may also be beneficial in cases of delayed hypersensitivity reactions. In conclusion, sea urchin stings are generally mild and may go unnoticed in many. However, neglected injuries and secondary infections can be notorious as it may lead to chronic joint complications. Hence, tissue culture must be sought, whenever secondary infections are suspected and early intervention with empiric antibiotics is advised for spines especially those near joints to avoid delayed complications.

## Conflicts of interest

None disclosed.
